# Italian Ketogenic Mediterranean Diet in Overweight and Obese Patients with Prediabetes or Type 2 Diabetes

**DOI:** 10.3390/nu14204361

**Published:** 2022-10-18

**Authors:** Cincione Raffaele Ivan, Antonietta Messina, Giuseppe Cibelli, Giovanni Messina, Rita Polito, Francesca Losavio, Ester La Torre, Vincenzo Monda, Marcellino Monda, Stefano Quiete, Elias Casula, Nicola Napoli, Giuseppe Defeudis

**Affiliations:** 1 Department of Clinical and Experimental Medicine, University of Foggia, 71122 Foggia, Italy; 2 Unit of Dietetics and Sports Medicine, Department of Experimental Medicine, Section of Human Physiology, Università degli Studi della Campania “Luigi Vanvitelli”, 80100 Naples, Italy; 3 Department of Medical and Surgical Sciences, University of Foggia, 71122 Foggia, Italy; 4 IRCCS Santa Lucia Foundation, 00128 Rome, Italy; 5 Unit of Endocrinology and Diabetes, Department of Medicine, University Campus Bio-Medico of Rome, 00128 Rome, Italy

**Keywords:** VLCKD_MED, VLCD_MED, Mediterranean diet, type 2 diabetes, prediabetes, overweight, obesity

## Abstract

Obesity is a multifactorial disease strongly associated with insulin resistance and/or type 2 diabetes mellitus. Correct nutrition represents a valid strategy to fight these dysmetabolic pathologies responsible for numerous diseases, including inflammatory and cardiovascular ones. Medical nutrition therapy, including a Mediterranean diet (MD) and a very low-calorie ketogenic diet (VLKCD), is the first-line treatment for prediabetes/diabetes and overweight/obesity. Eighty patients (forty women and forty men) affected by overweight/obesity and type 2 diabetes mellitus or impaired glucose tolerance or impaired fasting glucose (51 (ys) ± 1.75; BMI (kg/m^2^) 33.08 ± 1.93; HA1c (%): 6.8% ± 0.25) were enrolled at the University Service of Diet Therapy, Diabetology and Metabolic Diseases, Policlinico Riuniti Hospital of Foggia, and subjected to a very-low-calorie Mediterranean diet and a very-low-calorie ketogenic Mediterranean diet for thirty days. Both diets result in a marked decrease in body weight (kg) and BMI (kg/m^2^). At the same time, only the very-low-calories ketogenic Mediterranean diet reduced waist and hip circumferences. Both diets helped reduce fat mass, but a major loss was achieved in a very low-calorie ketogenic Mediterranean diet. Among gluco-metabolic parameters, only the very-low-calorie ketogenic Mediterranean diet group showed a significant decrease in fasting blood glucose and HbA1c, insulin, C-peptide total cholesterol, LDL, and triglycerides. The results of our study seem to show that the very-low-calorie ketogenic Mediterranean diet is a good strategy to improve rapidly metabolic, anthropometric, and body composition parameters in patients with prediabetes or diabetes and overweight/obesity.

## 1. Introduction

Obesity is a multifactorial disease and is the first cause of risk of developing insulin resistance and type 2 diabetes mellitus (T2DM) [1]. Prediabetes and type 2 diabetes mellitus (T2DM) have become widespread globally in recent decades [1], and it has now been widely demonstrated that the incidence of these pathologies is very high in overweight/obese patients [2]. Numerous studies show that the effects of obesity on glucose metabolism result in the deterioration of glucose tolerance, the development of insulin resistance and the consequent damage of the secretory function of beta-cells [2,3,4]. The incidence of obesity has tripled in the last 20 years, and currently, in European countries, 10–20% of men and 10–25% of women are obese (body mass index >30). There is growing evidence that one of the main trends in obesity is reduced physical activity levels and a lack of correct nutrition [5,6]. In this scenario, the role of lifestyle and nutrition appears to be fundamental. In particular, correct nutrition represents the most epigenetic factor applicable in short periods, and it has lasting effects. Indeed, research data have reported that caloric restriction reduces or slows the onset of diseases related to obesity such as T2DM, inducing considerable weight loss and having beneficial anti-inflammatory effects, reducing the production of free radicals, and favoring greater resistance to stress and a prolonged lifespan [7]. In light of this, a caloric restriction diet can be an efficient therapeutic approach to promote weight loss in obese, diabetic, and prediabetic patients. However, conflicting results have been reported regarding the ideal diet composition for nutritional therapy for these dysmetabolic diseases [8,9]. It is known that the Mediterranean diet has important beneficial effects as it is not just a diet but a real lifestyle; however, numerous studies have been conducted in recent years to investigate other types of nutritional interventions, such the ketogenic diet (KD) and the very low-calorie ketogenic diet [10], as medical nutrition therapy for diabetic disease and obesity [8,9,11,12,13,14]. The VLCKD has been recently proposed as a nutritional strategy in the multidisciplinary therapy of obese patients and other related gluco-metabolic and endocrine complications [15,16,17,18]. The benefits of the VLCKD have been demonstrated in body composition, metabolic profile, inflammation, oxidative stress, and obesity-related cardiovascular risk factors such as T2DM and hypertension in people with overweight/obesity [19,20,21].

The VLCKD provides 600–800 total kcal/day and is based on a significant reduction in daily carbohydrate intake, less than 50 g/day, about 10–13% of total energy intake, which simulates the physiologic state of fasting and increases ketone production and determines a state of ketosis [22,23]. The reduction in carbohydrates significantly decreases glycemia and insulin levels, elevating glucagon.

The low insulin: glucagon ratio induces the depletion of liver and muscle glycogen stores and activates neo-glycogenesis and lipolysis in adipose tissue and, consequently, the hepatic generation of ketone bodies acetoacetate, β-hydroxybutyrate, and acetone. Ketogenic diets induce a metabolic condition called “physiological ketosis,” which differs from pathological diabetic ketosis [24,25].

The VLCKD also includes a relative increase in the consumption of fats, about 44% of total calories, and protein, about 43%, with a daily protein intake ranging from 1.2 to 1.5 g/kg of the ideal body weight [21].

Ketone molecules exert an anorexic action by activating the ventromedial nucleus of the hypothalamus. This action combined with the satiating effects of proteins makes the KD more effective in controlling hunger and thus increases adherence to nutritional intervention [20].

The preservation of protein mass, obtained through daily protein intake, is another advantage of the ketogenic diet, especially compared to fasting and starvation [26]. So, ketogenic diets have healthier properties than conventional high carbohydrate/low-fat diets. Similarly, the Mediterranean diet has been shown to have several other health advantages.

In the light of this evidence, the purpose of this research was to test an innovative dietary strategy based on the syncretism between the nutritional model of the Mediterranean diet combined with the specific metabolic actions offered by the very-low-calorie ketogenic diet (or VLCKD_MED), compared with a very-low-calorie Mediterranean diet (or VLCD_MED), to obtain an improvement in gluco-metabolic control, body weight, and body composition in overweight and obese patients with prediabetes or T2D over a very short period of thirty days.

The diet approach used in this study is innovative, blending the Mediterranean diet and the ketogenic diet, using and enhancing the beneficial effects of both.

## 2. Materials and Methods

### 2.1. Patients

Eighty patients (forty women and forty men) affected by overweight/obesity and T2DM or prediabetes age (years): 51.95 ± 1.75; BMI (kg/m^2^): 33.08 ± 1.83; HA1c (%): 6.5% ± 0.25) were enrolled at the University Service of Diet Therapy, Diabetology and Metabolic Diseases, Policlinico Riuniti Hospital of Foggia, Puglia, Italy.

The 80 patients were divided into two groups of 40 and distributed as follows. In the VLCKD_MED group 20 patients was overweight and had a form of prediabetes, of which 5 women with impaired glucose tolerance and 5 women with impaired fasting glucose and 5 men with impaired fasting glucose and 5 men with impaired glucose tolerance while 20 patients was obese, 10 women and 10 men, and had type 2 diabetes. The VLCD_MED group had the same composition. Overweight patients with prediabetes and obese patients with type 2 diabetes mellitus were equally distributed between the two groups. The 50/50 gender balance in each group was predetermined before the start of the study.

The inclusion criteria were BMI (kg/m^2^) > 25, age > 18 years and < 65 years, their current diet consisting of at least 50% of their daily energy intake in carbohydrates, normal renal and hepatic function, overweight/obesity, and T2DM or impaired glucose tolerance or impaired fasting glucose. The exclusion criteria included: previous conditions of gout or hyperuricemia, pregnancy or breast-feeding absence of any other diseases other than T2DM, altered glucose tolerance, or impaired glucose tolerance. After starting the experimental protocol, all patients suspended oral hypoglycaemic agents. Participants were informed of the potential hazards and discomforts by written and oral information. Before taking part in the study, all patients provided written consent. The study was approved by Ethics Committee (Reference: CT22_2018), and it was conducted in accordance with the Declaration of Helsinki.

### 2.2. Experimental Design

Over thirty days, a two-arm parallel controlled not-randomized pilot trial was implemented to test the effects of a very-low-calorie ketogenic Mediterranean diet on glycol metabolic blood parameters, weight loss, and body composition compared with a very-low-calorie Mediterranean diet.

As the adopted study design is a small-scale pilot trial, no sample size was calculated. Retrospectively, a sample size based on the mean difference in HbA1c (%) and calculated SD observed in previous studies [27] identified a number of 15 participants per group as the sample size needed to detect a hypothetical treatment effect for a before-and-after study with a significance level of 5% and a study power of 80%. As a result, the number of patients participating in this study is statistically appropriate. Patients were divided into two groups of equal numbers, where one group followed a very-low-calorie ketogenic Mediterranean diet (VLCKD_MED group 40 patients, 20 men, 20 women) with a total carbohydrate intake below 50 g and a daily calorie intake set at around 800 kcal/day. The other group had a very-low-calorie Mediterranean diet (VLCD_MED group 40 patients, 20 men, 20 women) with 800 total daily kcal.

**Figure 1:**
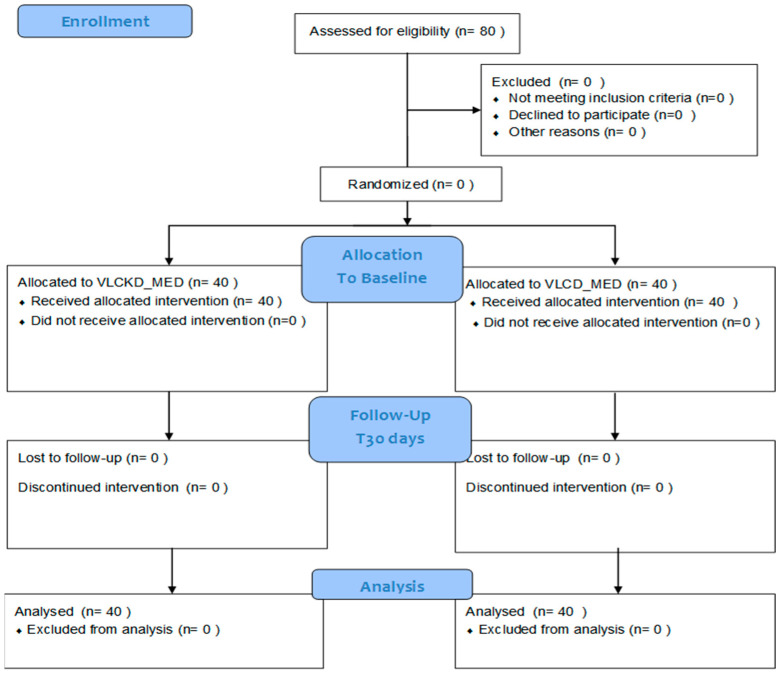
VLCKD_MED: very-low-calorie ketogenic Mediterranean diet and VLCD_MED: very-low-calorie Mediterranean diet.

Anthropometric, bio-impedance, and blood chemistry assessments were performed in the VLCKD_MED group and VLCD_MED group at the beginning of the study (T0) and after thirty days (T1) concurrently in each group. The experimental design is shown in Figure 1 as a CONSORT Flow Diagram.

### 2.3. Measurements

After verifying compliance with the inclusion criteria, anthropometric measures, bioelectrical impedance analysis (BIA), and urine and blood samples were collected before and at the end of the dietary program.

#### 2.3.1. Anthropometric and Bioelectrical Impedance Analysis Measures

The anthropometric characteristics were measured and are listed below: weight, height, body mass index, and waist and hip circumferences.

Bodyweight was measured at each visit on the same scale with the subject wearing light clothing, with shoes and socks removed, to the nearest 0.1 kg using an electronic scale with bioimpedance analysis (InBody 770. Co., Ltd., 625 Eonju-ro, Gangnam-gu, Seoul, Korea) and height was measured to the nearest 1 cm using a stadiometer (InBody BSM170 Co., Ltd., 625 Eonju-ro, Gangnam-gu, Seoul, Korea), body mass index was calculated as body weight (kg)/height^2^ (m)^2^, waist circumference and hips circumference were measured in centimeters with a flexible tape measure, respectively, as the smallest circumference between the lowest rib and the iliac crest on the midaxillary line, and at the level of the widest circumference over the great trochanters, where the waist to hip ratio was calculated as waist measurement divided by the hip measurement [28].

In each subject, the body composition analysis was assessed by bioimpedentiometry (InBody 770. Co., Ltd., 625 Eonju-ro, Gangnam-gu, Seoul, Korea). The following parameters were detected: body fat mass expressed in kilograms (FM), body fat mass expressed as a percentage (FM%), free fat mass expressed in kilograms (FFM), and total body water expressed in kilograms (TBW), where the basal metabolic rate was expressed in Kcal and calculated with the Harris–Benedict equation [14].

#### 2.3.2. Urine and Blood Samples

In addition, patients also underwent daily ß-Hydroxybutyrate tests on whole-capillary blood by finger test strips, based on an electrochemical measure and ß-hydroxybutyrate dehydrogenase as an enzyme system (glucomen areo 2K ß-Ketone Sensor, EN ISO 15197:2015, Menarini Diagnostics, Florence, Italy). Fasting venous blood and urine samples were collected and analyzed at the centralized laboratory of the Policlinico Riuniti University Hospital of Foggia for hepatic cytolysis indexes (GOT/AST GPT/ALT, GGT), glucose, glycosylated hemoglobin (Hb1Ac), total cholesterol, high-density lipoprotein cholesterol HDL, low-density lipoprotein cholesterol LDL, triacylglycerol, microalbuminuria, insulinemia, C peptide, and creatinine. Blood was collected in EDTA vacutainer tubes. A photometric assay analyzed a second blood sample for total cholesterol and triacylglycerols. The glucose oxidase technique was used to assess plasma glucose concentrations colorimetrically. HDL cholesterol was determined using an enzyme immunoassay. Creatinine was measured colorimetrically. LDL fraction was calculated from Friedewald’s formula [29]. Liver cytolysis was detected by spectrophotometry. The baseline characteristics of the two patient groups are reported in Table 1.

### 2.4. Diet Program

Patients independently prepared the two diets at home each day, using the following cooking methods: steam, boiling, cooking and grilling. Personal dietary intake was measured by weighted dietary diaries, compiled by patients and sent every five days by email to registered dieticians. In addition, every ten days, group meetings were held between the registered dieticians and patients to gather general feedback, assess diet adherence and provide help.

#### 2.4.1. Very-Low-Calorie Ketogenic Mediterranean Diet Characteristics

The very-low-calorie ketogenic Mediterranean diet was formulated according to the ketogenic diet criteria but adopting as many foods as possible from the Mediterranean diet. The aim was to obtain the condition of “Mediterranean” ketosis, induce weight loss, and improve gluco-metabolic parameters while preserving lean mass through an adequate amount of protein without configuring the diet as a high-protein diet.

For the very-low-calorie ketogenic Mediterranean diet, a protein intake of 1.2–1.4 g/kg/ideal body weight for men was used, while for women, the protein intake was 1.1–1.2 g/kg/ideal body weight.

The ideal body weight was calculated with the inverse formula of BMI (kg/m^2^) using a target value of BMI (kg/m^2^) of 22, so the proteins represented on average 32 percent of daily calories, with differences between the patients according to individual needs and body composition. The daily calorie intake was around 800 kcal/day, configuring a very-low-calorie ketogenic diet.

The maximum daily carbohydrate intake was set to less than 50 g to reduce blood glucose and insulin and obtain the hepatic production of ketones through the consequent rapid activation of lipolysis. Carbohydrate intake averaged 20 g per day, based on the number of vegetables consumed, and represented 10 percent of daily calories.

The amount of lipids was about 52 g, corresponding to 58 percent of daily calories of fat per day, most of which are of plant origin, also obtained from extra virgin olive oil and oleaginous fruits. The extra virgin olive oil amounted to 20 g consumed during the day, adding to the lipid contents of white meat and fish.

The breakfast consists of oleaginous fruit such as nuts, almonds, cashews, pistachios, and peanuts. Unsweetened soft drinks such as herbal teas, tea, and coffee were used as they do not interfere with the condition of ketosis and increase compliance with the diet of the enrolled patients.

A two-course meal was provided for lunch: one based on white meat or fish and a second course based on cooked or raw green vegetables. The patients could choose from two lists of vegetables.

The first list included the vegetables that the patient could eat in a maximum amount of 200 g per day, as the carbohydrate component of these vegetables, if consumed in larger amounts than those indicated, exceeds the daily limit of 50 g. The second list includes the vegetables that patients could eat in free quantities, given that their limited carbohydrate contents do not exceed the maximum daily carbohydrate limits.

With regard to white meat or fish, the patient could choose from a list of foods that varied daily, provided that fish was consumed at least four times a week. The portions of white meat or fish were assigned according to the individual protein and body composition requirements. White meat or fish can be replaced with eggs no more than two times a week, or tuna, squid, cuttlefish, or octopus.

Other than water, no less than 2 L per day, there was also an allowed amount of red wine equal to 150 mL per day, typical of the Mediterranean diet, which has remarkable antioxidant properties. Dinner follows the same pattern as lunch: a portion of white meat or fish and a portion of cooked or raw green vegetables with extra virgin olive oil. Wine is not allowed for dinner. Table 2 shows the VLCKD_MED food list.

The need for minerals, vitamins, and fibers is filled from the various vegetables and relative amount choices. In fact, patients of both dietary groups were encouraged to eat vegetables, especially those included in the free quantities list, and to exceed the recommended daily serving size, varying the choice and combination of the vegetables as much as possible [30]. In addition, all patients also took a daily (1 caplet each morning) multivitamin multimineral supplement [15] (containing Molybdenum 50 mcg, Magnesium 120 mg, Calcium 200 mg, Phosphorus 105 mg, Zinc 5 mg, Iron 3.75 mg, Iodine 100 mcg, Manganese 2 mg, Potassium 3 mg, Copper 0.5 mg, Chromium 40 mcg, Selenium 30 mcg, Niacin 20 mg (as niacin equivalents), Vitamin A 800 mcg, Folic Acid 200 mcg, Biotin 62.5 mcg, Vitamin C 80 mg, Vitamin E 24 mg (as alpha-tocopherol equivalent), vitamin K 30 mcg, Pantothenic Acid 7.5 mg, Vitamin B6 2.1 mg, Vitamin B2 2.1 mg, Vitamin B1 1.8 mg, Vitamin D3 10 mcg, and Vitamin B12, 3 mcg (multimineral multivitamin Multicentrum, GlaxoSmithKline S.p.A. Verona, Italy).

#### 2.4.2. Very-Low-Calorie Mediterranean Diet Characteristics

The very-low-calorie Mediterranean diet was formulated according to the Mediterranean diet’s typical macros, with a total daily energy intake of 800 Kcal, thus configuring a very-low-calorie diet, with 50 percent carbohydrates, 25 percent proteins, and 25 percent fats. The daily carbohydrate intake exceeded 50 g, so the hepatic production of ketones did not occur.

Unlike the VLCKD_MED diet, the VLCD_MED has no limitations in the choice of foods, where the total daily energy intake represents the only limitation. The lipids are of plant origin and are consumed through extra virgin olive oil, consumed during the day, and added to the lipid contents of white meat and fish.

The breakfast consists of coffee or tea with rusks or wholemeal bread. A two-course meal was provided for lunch: one based on grains or legumes and a second course based on cooked or raw vegetables. No lists of vegetables were provided from which the patient could choose.

For dinner, a two-course meal was provided with white meat or fish, varying daily, and fish was consumed at least four times a week with cooked or raw vegetables. The portions of white meat or fish were assigned according to the individual protein and body composition requirements. White meat or fish can be replaced with eggs no more than two times a week, or tuna, squid, cuttlefish, or octopus. The diet also included fresh fruit. In addition, all patients also took a daily (1 caplet each morning) multivitamin multimineral supplement (containing Molybdenum 50 mcg, Magnesium 120 mg, Calcium 200 mg, Phosphorus 105 mg, Zinc 5 mg, Iron 3.75 mg, Iodine 100 mcg, Manganese 2 mg, Potassium 3 mg, Copper 0.5 mg, Chromium 40 mcg, Sele-nium 30 mcg, Niacin 20 mg (as niacin equivalents), Vitamin A 800 mcg, Folic Acid 200 mcg, Biotin 62.5 mcg, Vitamin C 80 mg, Vitamin E 24 mg (as alpha-tocopherol equivalent), vitamin K 30 mcg, Pantothenic Acid 7.5 mg, Vitamin B6 2.1 mg, Vit-amin B2 2.1 mg, Vitamin B1 1.8 mg, Vitamin D3 10 mcg, and Vitamin B12, 3 mcg (multimineral multivitamin Multicentrum, GlaxoSmithKline S.p.A. Verona, Italy).

The exact composition of the very-low-calorie ketogenic Mediterranean and the very-low-calorie Mediterranean diet is reported in Table 3.

#### 2.4.3. Diets Scores

The Mediterranean diet serving score [31] was applied to calculate the adherence of the very-low-calorie ketogenic Mediterranean diet to the characteristics of the Mediterranean diet based on the Mediterranean food pyramid. This index ranges from 0 to 24 points for an adult and from 0 to 23 for a teenager; a score of 1, 2, or 3 is given for each item and 0 is given if the number of servings is lower or higher than the recommended ones. In adults, 1 point is assigned for wine, amounting to one glass for women and two for men. The higher the total score, the greater the adherence to the Mediterranean diet.

Although alcohol is not mandatory in the Mediterranean diet, the use of wine, particularly red wine, is advisable for the intake of polyphenols and resveratrol [32]. In addition, alcohol can increase conviviality and, therefore, adherence to the diet. Patients were strongly encouraged to favor the use of white meat and mainly fish as protein sources. Table 4 shows the Mediterranean Diet Serving Score based on the VLCKD_MED food list.

The Mediterranean adequacy index (MAI) [33] can be easily obtained by dividing the sum of the percentages of dietary energy contained in typical products of a healthy reference Mediterranean Italian diet or HRIMD (healthy reference Italian Mediterranean diet), by the sum of the percentages of dietary energy from the non-Mediterranean-products diet, according to the following equation:

$$\mathrm{MAI}=\frac{\left(\%\mathrm{energy}\;\mathrm{cereals}+\mathrm{legumes}+\mathrm{potatoes}+\mathrm{vegetables}+\mathrm{fresh}\;\mathrm{and}\;\mathrm{dry}\;\mathrm{fruit}+\mathrm{fish}+\mathrm{wine}+\mathrm{evo}\;\mathrm{oil}\right)}{\left(\%\mathrm{energy}\;\mathrm{milk}+\mathrm{cheese}+\mathrm{meat}+\mathrm{eggs}+\mathrm{animal}\;\mathrm{fat}\;\mathrm{and}\;\mathrm{margarine}+\mathrm{sweet}\;\mathrm{beverages}+\mathrm{cake}/\mathrm{pies}+\mathrm{cookies}\right)}$$

We also assessed the ORAC score [34] to measure the antioxidant action of the very-low-calorie ketogenic Mediterranean diet. This value depends mainly on the types of vegetables consumed by the patients. The average ORAC score of the vegetables was 1175 units; therefore, with two portions of vegetables per day of 200 g each, the ORAC units reached an average of 4.700 units, which is added to the ORAC value obtained from red wine and is variable, but rarely less than 500 per 100 g. Finally, with olive oil, we reach more than 400 units.

The PRAL [35] milliequivalents of acid per day were also calculated according to Remer [14] using the following formula: (0.49 × total protein intake) + (0.037 × phosphorus intake) − (0.021 × potassium intake) − (0.026 × magnesium intake) − (0.013 × calcium intake) as a measurement of the amount of dietary acid load of the VLCKD_MED.

### 2.5. Statistical Analysis

All the data were analyzed using SPSS version 22 (SPSS Inc., Chicago, IL, USA). Prior to undergoing parametric or non-parametric statistical procedures, assumption of normality distribution of data was assessed with Shapiro–Wilks’ test; assumption of homoscedasticity was assessed with Levene’s test.

The effect of VLCKD_MED and VLCD_MED was assessed in the following dependent variables: weight (kg), BMI (kg/m^2^) FM (kg), FFM (kg), TBW (kg), CV (cm), CF (cm), WHR (ratio) and MB (kcal) by comparing the % change values before and after the VLCKD_MED and the VLCD_MED diet groups. This analysis was performed by a MANCOVA with “diet” (VLCKD_MED vs. VLCD_MED) as a between-patients factor; in addition, since we were interested in the possible influence of the sex and age of each patient, we inserted these two variables as a factor and covariate in the same MANCOVA. In case of violations of parametric assumptions, we used the Pillai’s trace as a multivariate statistic. Level of significance was set at α = 0.05. To reduce the occurrence of type I errors, all the *p*-values were corrected with the Sidak method.

The effect of VLCKD_MED and VLCD_MED diets was also assessed in the following dependent variables—blood glucose (mg/dL), insulin (µU/mL), glycosylated haemoglobin (%), HOMA index, C peptide (ng/mL), total cholesterol (mg/dL), HDL cholesterol (mg/dL), LDL cholesterol (mg/dL), triglyceridemia (mg/dL), GOT glutamic oxaloacetic transaminase (U/L), GPT glutamic pyruvic transaminase (U/L), GGT gamma-glutamyl transferase (U/L) and creatinine (mg/dL)—by comparing the % change values before and after the VLCKD_MED and the VLCD_MED diet groups. This analysis was performed by a MANCOVA with “diet” (VLCKD_MED vs. VLCD_MED) as a between-patients factor; in addition, since we were interested in possible influence due to the sex and age of each patient, we inserted these two variables as a factor and covariate in the same MANCOVA. In case of violation of parametric assumptions, we used the Pillai’s trace as a multivariate statistic. Level of significance was set at α = 0.05. To reduce the occurrence of type I errors, all the *p*-values were corrected with the Sidak method.

## 3. Results

We had achieved a total compliance of patients and the absence of dropouts. This was determined by a pre-selection of patients, which allowed us, even before starting the study, to identify the patients that were most motivated to follow the diet regimen. So, patients were chosen for eligibility, and eligibility was correlated with diet compliance; therefore, this research is not a random population study.

### 3.1. Anthropometrical and Body Composition Results

Raw values of all the dependent demographic, anthropometric, and body composition variables before and after the VLCKD_MED and VLCD_MED diets are reported in Table 5.

**Figure 2:**
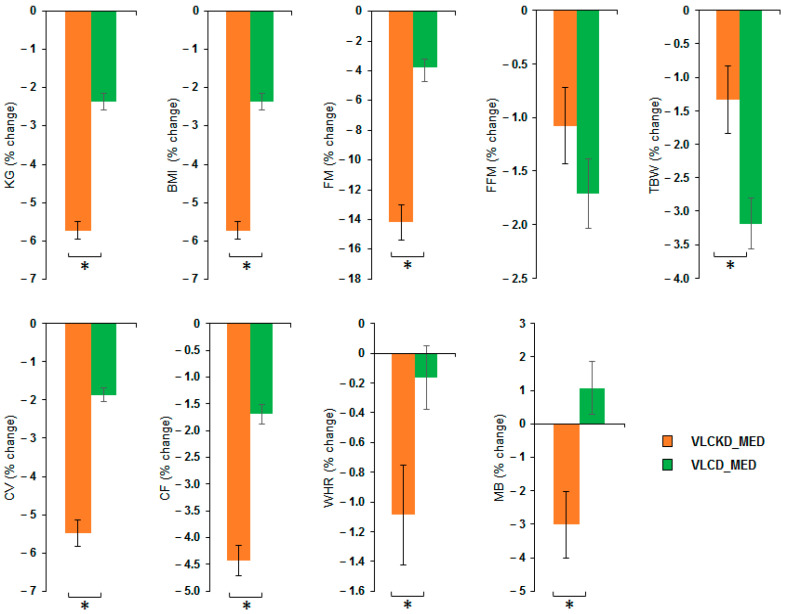
Percentage change of the anthropometrical and body composition variables after VLCKD_MED and VLCD_MED diet. The histogram depicts the % change (post-pre diet) in the level of weight (kg), BMI: body mass index (kg/m^2^), FM: fat mass (kg), FFM: free fat mass (kg), TBW: total body water (kg), CV: waist circumference (cm), CF: hip circumference (cm), WHR: waist to hip ratio; MB: basal metabolism (kcal) for the group following the VLCKD_MED diet (orange bars) and VLCD_MED diet (green bars). Error bars depict the standard error of the mean. * depicts a significant difference, *p* < 0.05.

MANOVA of VLCKD_MED and VLCD_MED effects showed a significant effect of the diet factor (V = 0.852; F(8,36) = 25.836; *p* < 0.001; η2 = 0.852) but no effect of the sex factor (V = 0.345; F(8,36) = 0.2.375; *p* = 0.056; η2 = 0.345) nor of covariate age (V = 0.203; F(8,36) = 1.146; *p* = 0.358; η2 = 0.203). Univariate ANOVAs showed that the diet factor has a significant effect on the following variables: weight (kg) (F(1,43) = 126.4; *p* < 0.001; η2 = 0.746); BMI (kg/m^2^) (F(1,43) = 126.4; *p* < 0.001; η2 = 0.746); FM (kg) (F(1,43) = 102.417; *p* < 0.001; η2 = 0.704); TBW (kg) (F(1,43) = 7.980; *p* = 0.007; η2 = 0.157); CV (cm) (F(1,43) = 93.354; *p* < 0.001; η2 = 0.685); CF (cm) (F(1,43) = 70.365; *p* < 0.001; η2 = 0.621); WHR (ratio) (F(1,43) = 5.036; *p* = 0.03; η2 = 0.105) and MB (kcal) (F(1,43) = 10.965; *p* = 0.002; η2 = 0.203). No difference between the diets was observable for the variable FFM (kg) (F(1,43) = 1.974; *p* = 0.167; η2 = 0.044). Post hoc analyses showed that VLCKD_MED caused a stronger reduction, compared to the VLCD_MED, in the levels of body weight (kg) (post hoc *p* < 0.001); BMI (kg/m^2^) (post hoc *p* < 0.001); FM (kg) (post hoc *p* < 0.001); TBW (kg) (post hoc *p* = 0.007); CV (cm) (post hoc *p* < 0.001); CF (cm) (post hoc *p* < 0.001); WHR (ratio) (post hoc *p* = 0.030); and MB (kcal) (post hoc *p* = 0.002). The anthropometrical and body composition results comparison as % change in the anthropometrical and body composition variables after VLCKD_MED and VLCD_MED diet is reported in Figure 2.

### 3.2. Diet Score Results

To confirm the compliance of the two diets with the Mediterranean nutritional style, the specific and typical food choices were also checked through indices such as the Mediterranean diet serving score and the Mediterranean adequacy index.

Moreover, to assess the specific health benefits of the diet protocol, the oxygen radical absorbance capacity, or ORAC, and the potential for renal acid load, or PRAL, scores have been evaluated in addition.

The Mediterranean diet serving calculated for the very-low-calorie ketogenic Mediterranean diet has a value of twelve, which is therefore within the reference range. The Mediterranean diet serving score computed for the very-low-calorie Mediterranean diet has a higher score, equal to sixteen, thanks to the cereals, legumes, fresh fruits, and all types of vegetables included in it, so it is different compared with VLCKD_MED.

The current average value of the Mediterranean adequacy index, also known as MAI HRIMD, is between 4.0 and 8.5, presenting variations depending on food intake patterns. Therefore, the higher the value of the MAI, the more the energy source comes from Mediterranean foods. The MAI value of the very-low-calorie ketogenic Mediterranean diet was 5.083, comfortably within the above reference range, whereas the value of the MAI of the very-low-calorie Mediterranean diet was 9.

The total ORAC score was higher than 5000 units, confirming the Mediterranean antioxidant properties of VLCKD_MED. On the other hand, the ORAC score of VLCD_MED was higher than 8000 units because of the absence of limitations in the choice of vegetables, cereals and fruits.

The PRAL of the VLCKD_MED diet, and even more so of the VLCD_MED diet, has a negative value depending on the different vegetable contents, which gives the diets strongly alkaline characteristics and attests that the VLCKD_MED diet has a normal proteic and phosphoric load. Negative PRAL values indicate that both diets are alkaline.

### 3.3. Blood Exams Results

Raw values of all the hematochemical dependent variables before and after the VLCKD_MED and VLCD_MED diets are reported in Table 6.

MANOVA of the VLCKD_MED and VLCD_MED diet effects showed a significant effect of the diet factor (V = 0.964; F(13,31) = 64.265; *p* < 0.001; η2 = 0.964) but no effect of sex factor (V = 0.198; F(13,31) = 0.588; *p* = 0.845; η2 = 0.198), or of the covariate age (V = 0.354; F(13,31) = 1.307; *p* = 0.261; η2 = 0.354). Univariate ANOVAs showed that the diet factor has a significant effect on the following variables: GLYCEMIA, i.e., blood glucose (mg/dL) (F(1,43) = 13.210; *p* = 0.001; η2 = 0.235); INS, i.e., insulin (µU/mL), (F(1,43) = 103.419; *p* < 0.001; η2 = 0.706); HB_GLIC, i.e., glycosylated hemoglobin (%) (F(1,43) = 475.560; *p* < 0.001; η2 = 0.917); HOMA index (F(1,43) = 43.716; *p* < 0.001; η2 = 0.504); PEP_C, i.e., C-peptide (ng/mL) (F(1,43) = 62.807; *p* < 0.001; η2 = 0.594); COL_TOT, i.e., total cholesterol (mg/dL) (F(1,43) = 74.747; *p* < 0.001; η2 = 0.634); COL_LDL cholesterol (mg/dL) (F(1,43) = 24.914; *p* < 0.001; η2 = 0.367); TRIG, i.e., triglyceridemia (mg/dL) (F(1,43) = 22.853; *p* < 0.001; η2 = 0.347) and GPT glutamic-pyruvic transaminase (U/L) (F(1,43) = 22.230; *p* < 0.001; η2 = 0.341). No difference between the diets was observable for the variables COL_HDL cholesterol (mg/dL) (F(1,43) = 0.631; *p* = 0.431; η2 = 0.014); GOT, i.e., glutamic oxaloacetic transaminase (U/L) (F(1,43) = 1.888; *p* = 0.177; η2 = 0.042); GGT, i.e., gamma-glutamyl transferase (U/L) (F(1,43) = 0.365; *p* = 0.549; η2 = 0.008) and CREAT creatinine (mg/dL) (F(1,43) = 2.629; *p* = 0.112; η2 = 0.058).

**Figure 3:**
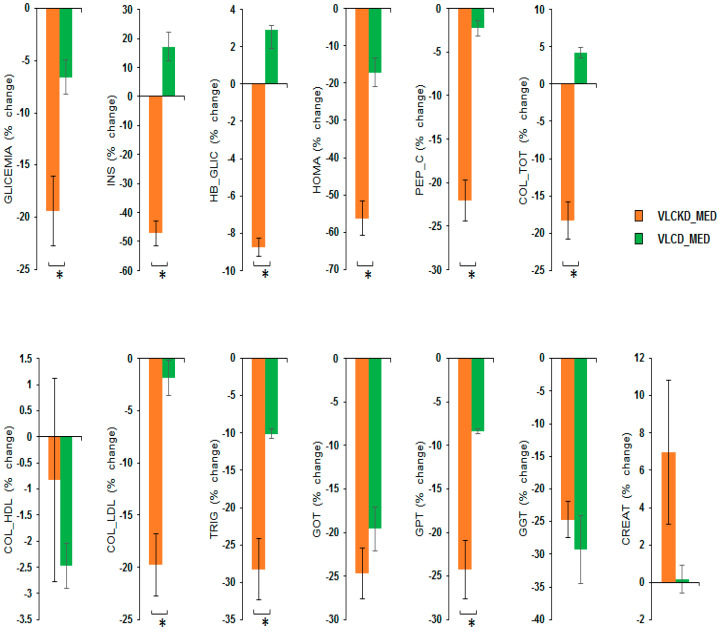
Percentage change in the blood chemistry variables after VLCKD_MED and VLCD_MED diet. The histogram depicts the % change (post-pre diet) in the level of: GLYCEMIA, i.e., blood glucose (mg/dL); INS, i.e., insulin (µU/mL); HB_GLIC, i.e., glycosylated hemoglobin (%); HOMA index; PEP_C, i.e., C-peptide (ng/mL); COL_TOT, i.e., total cholesterol (mg/dL); COL_LDL cholesterol (mg/dL); COL_HDL cholesterol (mg/dL) TRIG, i.e., triglyceridemia (mg/dL); GPT glutamic pyruvic transaminase (U/L), GOT, i.e., glutamic oxaloacetic transaminase (U/L); GGT, i.e., gamma-glutamyl transferase (U/L) CREAT creatinine (mg/dL) for the group following the VLCKD_MED diet (orange bars) and VLCD_MED diet (green bars). Error bars depict the standard error of the mean. * depicts a significant difference, *p* < 0.05.

Post hoc analyses showed that the VLCKD_MED diet caused a stronger reduction compared to the VLCD_MED diet in the levels of GLYCEMIA, i.e., blood glucose (mg/dL) (post hoc *p* = 0.001); INS, i.e., insulin (µU/mL) (post hoc *p* < 0.001); HB_GLIC, i.e., glycosylated hemoglobin (%) (post hoc *p* < 0.001); HOMA index (post hoc *p* < 0.001); PEP_C, i.e., C peptide (ng/mL) (post hoc *p* < 0.001); COL_TOT, i.e., total cholesterol (mg/dL) (post hoc *p* < 0.001); COL_LDL cholesterol mg/dL (post hoc *p* < 0.001); TRIG, i.e., triglyceridemia (mg/dL) (post hoc *p* < 0.001); and GPT glutamic pyruvic transaminase (U/L) (post hoc *p* < 0.001). A comparison of the blood chemistry variables as the % change in the blood chemistry variables after the VLCKD and VLCD_MED diet are reported in Figure 3.

## 4. Discussion

The main findings of the present study may be summarized in terms of a decrease in weight (kg) and BMI (kg/m^2^) and in a positive remodulation of metabolic parameters other than an improvement in body composition parameters. The results suggest that the very-low-calorie ketogenic Mediterranean diet, or VLCKD_MED, caused a significant and more extensive decrease compared to the very-low-calorie Mediterranean, or VLCD_MED, diet, in body weight, and also produced an improvement in body composition parameters and several metabolic markers linked to prediabetes, type 2 diabetes and risk of cardiovascular disease.

Other authors have successfully studied the Mediterranean ketogenic diet. In 2008, Guisado et al. [36] proposed the first Spanish ketogenic Mediterranean diet as an efficient method for reducing body fat, improving non-atherogenic lipid profiles, lowering blood pressure and optimizing fasting glucose levels, and as an effective and safe way to treat patients suffering from metabolic syndrome and associated nonalcoholic fatty liver disease. More recently, in 2011, Paoli et al. [37] proposed a modified Mediterranean ketogenic diet, defined as the KEMEPHY diet, achieving good results in reducing body weight and cardiovascular risk factors in overweight and obese patients.

However, both Mediterranean ketogenic diets have different characteristics. The Spanish ketogenic Mediterranean diet is an unlimited-calorie diet. The KEMEPHY Mediterranean ketogenic with phytoextracts diet protocol had a daily calorie content between 1000 and 1200 Kcal and provided the use of phytocompounds, with beta-adrenergic action stimulating lipolysis. So, both approaches, though developed as Mediterranean ketogenic diets, are not very-low-calorie ketogenic diet and, in the case of Paoli, include the use of herbal extracts that can enhance lipolysis regardless of nutritional intervention.

Conversely, the proposed dietary regimen is an exclusively nutritional intervention and is a very-low-calorie ketogenic Mediterranean diet [38,39]

Moreover, according to the previously published research, the reduction in appetite resulted from ketone production, and the higher effect on satiety is due to the use of proteins, which are linked to appetite control hormones such as ghrelin, amylin, and leptin [40,41,42]. For this purpose, capillary ß-hydroxybutyrate concentration was measured as a direct and reliable index of the occurrence of ketosis. If an increase in the blood concentration of ketones had not been detected, the purpose of the diet to induce the ketosis process would not have been achieved.

The absence of significant variations in the blood concentrations of the hepatic function indices, GOT (U/L), GPT (U/L), GGT (U/L), and serum creatinine allowed us to exclude any adverse changes in hepatic and renal function due to both dietary interventions.

Furthermore, satisfying results were obtained with VLCKD_MED compared to the VLCD_MED diet in improving the anthropometric and body compositions bioimpedance parameters, significantly and more intensely reducing body weight (kg), waist circumference (cm), hip circumference (cm), BMI (kg/m^2^), WHR (ratio) FM (kg), basal metabolism (kcal), and total body water (kg), with no significant differences in FFM (kg). However, the reduction in the basal metabolism and the total body water pre- and post-diet for both diets, considered as numerical absolute magnitude values, are clinically insignificant.

Additionally, VLCKD_MED, compared to the VLCD_MED diet, showed a substantial improvement with a significantly stronger reduction in the levels of fasting blood glucose (mg/dL), insulin (µU/mL), glycosylated hemoglobin (%), HOMA index, C peptide (ng/mL), total cholesterol (mg/dL), LDL cholesterol (mg/dL), triglyceridemia (mg/dL), and aspartate aminotransferase (U/L), with no difference between the diets for HDL cholesterol (mg/dL), alanine aminotransferase (U/L), gamma-glutamyl transferase (U/L), and serum creatinine (mg/dL). Interestingly, there is a significant reduction in C-peptide (ng/mL). This result suggests, together with the reduction in HOMA index, an improvement in insulin resistance that is strictly connected to a well-balanced gluco-metabolic condition.

The proposed nutritional treatment has also been effective in influencing blood lipoprotein values, with a significant decrease in total cholesterol (mg/dL), LDL cholesterol (mg/dL), and triglycerides (mg/dL), without a substantial reduction in the value of HDL (mg/dL). These results positively impact lipid profile control, slowing down or even stopping prediabetes and diabetes complications such as cardiovascular [43] or microvascular diseases [44].

Otherwise, the very-low-calorie Mediterranean diet shows a substantially smaller decrease in all anthropometric, body composition and gluco-metabolic parameters and therefore has not led to an unbalanced gluco-metabolic and lipidic control.

Despite the very low caloric intake, both diets have no FFM (kg) reduction. This is probably correlated with protein intake, which exerted a protein sparing effect on muscle mass and inhibited muscle catabolism, as already documented by other publications [45,46].

This pilot study was conducted in preparation for a larger successful future RCT to explore the efficiency and internal validity of the proposed trial.

Moreover, this study is an absolute novelty for the type of nutritional intervention adopted, a method of intervention that is currently very poorly investigated in the scientific medical literature, namely the VLCKD_MED.

The duration of only thirty days of the nutritional intervention has been determined in consideration of the following elements:

- The production kinetics of blood beta butyrate hydroxy reaches almost the maximum concentration value on the twentieth day, as demonstrated by Cahill and Veech [47];
- The lipolysis rate begins on the tenth day and reaches a plateau on the twentieth [48];
- The short duration ensures maximum patient compliance and avoids patients dropping out during the very-low-calorie ketogenic Mediterranean diet.

Even if the diet period was only very short, combining the very-low-calorie ketogenic diet with the Mediterranean diet means selecting the best elements of the two diets. The fusion of the two types of diet leads to effects on fasting blood glucose, lipid profiles, and rapid weight and fat loss, with the preservation of lean mass and with total compliance of patients. The patients benefit at the same time from the protective nutritional, anti-inflammatory, and antioxidant properties deriving from Mediterranean foods such as vegetables, oleaginous fruit, extra virgin olive oil, and wine [49], and as confirmed by the total ORAC and PRAL values, along with the scores of the Mediterranean diet serving and Mediterranean adequacy index.

## 5. Conclusions

We have demonstrated that the VLCKD_MED, compared with the VLCD_MED diet, exerts a stronger positive effect not only on anthropometric and body composition parameters, but also and most especially on gluco-metabolic and lipidic control, even if over a short time of only thirty days, in overweight/obese patients with prediabetes or non-insulin-dependent T2D, without any worsening of hepatic or kidney function.

The novelty of our approach is to have combined the many health advantages of the Mediterranean diet, with the positive effects of the state of “nutritional ketosis” to improves gluco-metabolic and lipid control, reduce body weight and change the body composition and consequently reduce cardio-vascular risk factors, of in overweight/obese people with prediabetes or T2D.

Finally, to the best of our knowledge, this study is the first exclusively nutritional intervention conducted in Italy based on the very-low-calorie ketogenic diet combined with the Mediterranean diet, without phytoextracts or drugs, over a very short time period, to testify to and confirm the effectiveness of the method discussed in this work, but further studies are needed to clarify the potential use of this diet approach in preventing and/or treating prediabetes or diabetes and overweight/obesity.

## Figures and Tables

**Table 1:** Baseline characteristics of patients.

	All Patients Mean (±sd)	VLCKD_MED Group Mean (±sd)	VLCD_MED Group Mean (±sd)	*p* Value
	N = 80	N = 40	N = 40	
Men/women	40/40	20/20	20/20	0.77
Age (years)	51.95 ± 1.75	52.08 ± 1.71	51.83 ± 1.80	0.092
Weight (kg)	90.37 ± 2.76	91.13 ± 2.79	89.62 ± 2.74	0.70
Height (m)	1.66 ± 0.02	1.65 ± 0.02	1.66 ± 0.02	0.78
BMI (kg/m^2^)	33.08 ± 1.83	33.42 ± 0.93	32.64 ± 0.98	0.56

Data are expressed as mean ± standard deviation (sd). *p* < 0.05.

**Table 2:** VLCKD_MED food list.

Oleaginous Fruit: Almonds, Nuts, Peanuts, Cashews, Hazelnuts, Pistachios
Fish: Sea bream, tuna, mullet, trout, swordfish, perch, sea bass, mackerel, cod, pike, dogfish, sole, squid, cuttlefish, octopus
White meat: Chicken, turkey, rabbit
Vegetables in unlimited quantities: All leafy vegetables: (Lettuce, Valerian, Belgian Salad, Arugula, Chicory, Endive, Escarole), Chard, Broccoli, Thistles, Cauliflower, Cabbage, Cucumber, Turnip Greens, Zucchini Flowers, Fennel, Mushrooms, Bean Sprouts, Green Peppers, Radish, Red Radicchio, Green Radicchio, Celery, Spinach, Zucchini
Vegetables maximum 200 g per day: Asparagus, Eggplant, Artichokes, Brussels Sprouts, Green Beans, Tomatoes, Red Peppers, Turnips, Yellow Pumpkin
Extra virgin olive oil

**Table 3:** VLCKD_MED and VLCD_MED composition.

Diet	VLCKD_MED		VLCD_MED	
Energy_Kcal/day_	800 Kcal/day		800 Kcal/day	
Fat g/day_% total daily energy	52 ± 3 g	58.25%	23 ± 6 g	25%
Protein g/day_% total daily energy	63 ± 5 g	31.75%	50 ± 8 g	25%
Carbohydrate g/day_% total daily energy	20 g	10%	100 g	50%

**Table 4:** VLCKD_MED: the Mediterranean Diet Serving Score.

Very Low-Calorie Ketogenic Mediterranean Diet	Recommendation	Score
Vegetables	≥2 servings/main meal	3
Olive Oil	1 serving/main meal	3
Nuts	1–2 servings/day	2
Eggs	2–4 servings/week	1
Fish	≥2 servings/week	1
White meat	2 servings/week	1
Red wine	1–2 glasses/day	1
	Total score	12

**Table 5:** Demographic, anthropometric, and body composition variables of the VLCKD_MED and VLCD_MED groups.

Variable (Mean ± SE)	Pre VLCKD_MED	Post VLCKD_MED	Pre VLCD_MED	Post VLCD_MED
Age (years)	52.08 ± 1.71	-	51.83 ± 1.80	-
Sex M/F	20/20	20/20	20/20	20/20
Height (m)	1.65 ± 0.02	-	1.66 ± 0.02	-
Body weight (kg)	91.13 ± 2.79	85.88 ± 2.599	89.62 ± 2.74	87.5 ± 2.66
BMI (kg/m^2^)	33.42 ± 0.93	31.52 ± 0.90	32.64 ± 0.98	31.87 ± 0.97
FFM (kg)	34.83 ± 2.01	30.24 ± 1.96	34.38 ± 1.99	33.23 ± 2.03
FFM (kg)	56.24 ± 2.16	55.65 ± 2.15	55.45 ± 2.16	54.54 ± 2.17
TTW (kg)	40.63 ± 1.56	40.04 ± 1.49	39.66 ± 1.55	38.4 ± 1.49
CV (cm)	107.60 ± 2.16	101.71 ± 2.06	104.5 ± 2.27	102.54 ± 2.22
CF (cm)	117.12 ± 2.12	111.96 ± 2.12	114.58 ± 2.04	112.64 ± 2.04
WHR (ratio)	0.92 ± 0.01	0.91 ± 0.01	0.91 ± 0.02	0.912 ± 0.02
MB (kcal)	1708.46 ± 61.79	1649.25 ± 50.67	1657.79 ± 60.12	1672.41 ± 58.19

FFM: free fat mass (kg); TBW: total body water (kg), MB: basal metabolism (kcal); BMI: body mass index (kg/m^2^); CV: waist circumference (cm); CF: hip circumference (cm); WHR: waist to hip ratio (ratio); “-” no change to the previous paired value.

**Table 6:** Blood chemistry dependent variables before and after the VLCKD_MED and VLCD_MED.

Variable (Mean ± SE)	Pre VLCKD_MED	Post VLCKD_MED	Pre VLCD_MED	Post VLCD_MED
Age (years)	52.08 ± 1.71	-	51.83 ± 1.80	-
Sex M/F	20/20	20/20	20/20	20/20
Height(m)	1.65 ± 0.02	-	1.66 ± 0.02	-
Glycemia (mg/dL)	134.79 ± 8.62	103.375 ± 3.54	127.41 ± 8.79	117.45 ± 7.35
INS (µU/mL)	20.16 ± 1.72	9.57 ± 0.70	20.42 ± 2.44	18.01 ± 2.21
HB_GLIC (%)	6.81 ± 0.24	6.20 ± 0.21	6.81 ± 0.26	6.61 ± 0.25
HOMA index	7.12 ± 1.02	2.49 ± 0.23	7.32 ± 1.54	5.76 ± 1.12
PEP_C (ng/mL)	2.91 ± 0.13	2.26 ± 0.11	2.73 ± 0.12	2.65 ± 0.11
COL_TOT (mg/dL)	205.33 ± 7.41	165.83 ± 6.19	214.87 ± 6.27	206.03 ± 5.61
COL_HDL (mg/dL)	51.04 ± 2.24	50.12 ± 1.95	52.12 ± 1.90	50.87 ± 1.92
COL_LDL (mg/dL)	120.08 ± 7.03	95.39 ± 5.77	127.01 ± 6.70	122.89 ± 5.85
TRIG (mg/dL)	171.04 ± 33.04	101.58 ± 7.92	178.66 ± 28.43	161.33 ± 25.91
GOT(U/L)	30.79 ± 3.17	22.62 ± 2.47	30.62 ± 3.34	25.21 ± 3.17
GPT (U/L)	39.96 ± 5.96	28.66 ± 3.94	31.25 ± 22.78	28.61 ± 4.27
GGT (U/L)	37.04 ± 4.22	26.95 ± 2.78	35.25 ± 3.64	29.19 ± 3.39
CREAT (mg/dL)	0.78 ± 0.03	0.81 ± 0.03	0.73 ± 0.03	0.72 ± 0.03

GLYCEMIA, i.e., blood glucose (mg/dL); INS, i.e., insulin (µU/mL); HB_GLIC, i.e., glycosylated hemoglobin (%); HOMA index; PEP_C, i.e., C-peptide (ng/mL); COL_TOT, i.e., total cholesterol (mg/dL); COL_LDL cholesterol (mg/dL); COL_HDL cholesterol (mg/dL) TRIG, i.e., triglyceridemia (mg/dL); GPT glutamic pyruvic transaminase (U/L), GOT, i.e., glutamic oxaloacetic transaminase (U/L); GGT, i.e., gamma-glutamyl transferase (U/L) CREAT creatinine (mg/dL). “-” no change to the previous paired value.

## Data Availability

The data used to support the findings of this study are available from the corresponding author upon request.
